# Urinary Hypaphorine as a Candidate Marker of FFQ-Derived Legume Intake Within the EAT-Lancet Framework in Free-Living Adults

**DOI:** 10.3390/foods15183279

**Published:** 2026-09-17

**Authors:** Teresa Cepeda-Vidal, João Tomé-Carneiro, Alberto Valdés, Nieves Rocío Colás-Ruiz, Edwin Fernández-Cruz, Isabel Espinosa-Salinas, Pilar Matía-Martín, Ana Barabash-Bustelo, Angélica Larrad-Sainz, Miguel Ángel Rubio-Herrera, Alfonso Luis Calle-Pascual, Alberto Cepeda-Sáez, J. Alfredo Martínez

**Affiliations:** 1 Laboratorio de Higiene, Inspección y Control de Alimentos (LHICA), Departamento de Química Analítica, Nutrición y Bromatología, Universidad de Santiago de Compostela, 15705 Santiago de Compostela, Spain; teresa.cepeda@rai.usc.es (T.C.-V.); alberto.cepeda@usc.es (A.C.-S.); 2 Food Bioactive Ingredients, Instituto Madrileño de Estudios Avanzados (IMDEA) Nutrition, Carretera de Cantoblanco 8, 28049 Madrid, Spain; 3 Metabolomics Platform, Institute of Food Science Research (CIAL, CSIC-UAM), Calle Nicolás Cabrera 9, 28049 Madrid, Spain; a.valdes@csic.es (A.V.); rocio.colas@csic.es (N.R.C.-R.); 4 Foodomics Laboratory, Institute of Food Science Research (CIAL, CSIC-UAM), Calle Nicolás Cabrera 9, 28049 Madrid, Spain; 5 Faculty of Life and Natural Sciences, Nebrija University, 28240 Madrid, Spain; efernanc@nebrija.es; 6 GENYAL Platform, Instituto Madrileño de Estudios Avanzados (IMDEA) Nutrition, Carretera de Cantoblanco 8, 28049 Madrid, Spain; mariaisabel.espinosa@nutricion.imdea.org; 7 Endocrinology and Nutrition Department, Hospital Clínico San Carlos, Instituto de Investigación Sanitaria San Carlos (IdISSC), 28040 Madrid, Spain; mmatia@ucm.es (P.M.-M.); ana.barabash@salud.madrid.org (A.B.-B.); angelica.larrad@salud.madrid.org (A.L.-S.); marubioh@gmail.com (M.Á.R.-H.); alfonsca@ucm.es (A.L.C.-P.); 8 Department of Medicine, Universidad Complutense de Madrid, 28040 Madrid, Spain; 9 CIBER Diabetes y Enfermedades Metabólicas Asociadas (CIBERDEM), Instituto de Salud Carlos III (ISCIII), 28029 Madrid, Spain; 10 Cardiometabolic Precision Nutrition Program, Instituto Madrileño de Estudios Avanzados (IMDEA) Nutrition, Carretera de Cantoblanco 8, 28049 Madrid, Spain; jalfredo.martinez@nutricion.imdea.org; 11 Centre of Medicine and Endocrinology, University of Valladolid, 47002 Valladolid, Spain; 12 Biomedical Research Centre for Obesity Physiopathology and Nutrition Network (CIBEROBN), Instituto de Salud Carlos III (ISCIII), 28029 Madrid, Spain

**Keywords:** hypaphorine, legumes, dietary intake biomarker, targeted metabolomics, leukocytes, nutritional epidemiology, food-frequency questionnaire

## Abstract

Dietary assessment is affected by recall, reporting, portion-size error, and food-composition inconsistencies. Hypaphorine, a tryptophan-derived betaine alkaloid found in pulses and other foods, has been proposed as a marker of legume exposure, but its performance in free-living populations remains uncertain. In this cross-sectional study of 138 adults, dietary intake was assessed by food-frequency questionnaire, and legume intake was represented by an FFQ-derived variable within the EAT-Lancet food-group framework. Urinary hypaphorine was quantified by targeted hydrophilic-interaction liquid chromatography-mass spectrometry. Associations were examined using Spearman correlations and multivariable linear models with HC3 robust standard errors. Predictive performance was assessed using repeated 10-fold cross-validation. Hypaphorine correlated with legume intake (*ρ* = 0.240; *p* = 0.0047). After adjustment for age, sex, BMI, energy intake, and log10-transformed urinary creatinine signal, each 10 g/day increment in legume intake was associated with 19.1% higher hypaphorine (95% CI, 7.2–32.2%; *p* = 0.0014). Estimation of the FFQ-derived legume-intake variable was limited (cross-validated R^2^ = 0.061), whereas discrimination between intake above and at or below the sample median was modest (AUC = 0.653). In the exploratory clinical screen, the inverse correlation with leukocyte count did not remain below the 0.05 FDR threshold (*q* = 0.090). A focused adjusted model yielded an inverse association (*β* = −1.38 × 10^3^/µL; *p* = 0.030), but exclusion of the most influential observation attenuated statistical support (*p* = 0.061). Urinary hypaphorine may provide modest information for ranking FFQ-derived legume exposure but should not be considered a quantitative measure of individual intake without further validation.

## 1. Introduction

Legumes, particularly pulses such as lentils, chickpeas, beans, and peas, provide plant protein, dietary fibre, micronutrients, and low-glycaemic-index carbohydrates, and regular consumption has been associated with favourable cardiometabolic outcomes, although the strength of evidence varies by outcome and population [1,2]. Legumes also occupy a central position in dietary frameworks that seek to align human health with environmental sustainability [3]. Accurate assessment of habitual legume exposure is therefore relevant to nutritional surveillance and intervention research.

Food-frequency questionnaires (FFQs), food records, and dietary recalls remain the principal tools for estimating usual intake, but they are affected by memory, reporting behaviour, portion-size estimation, and food-composition assumptions [4,5]. Biomarkers of food intake can complement self-report by providing an objective biological measure of exposure, and metabolomics has expanded the discovery of food-derived signals in urine and blood [6,7]. Recent work within the Dietary Deal project has similarly explored urinary and biochemical markers for classifying food-group intake and dietary quality [8,9].

Hypaphorine (tryptophan betaine) is a dietary indole alkaloid detected in legumes, including lentils, chickpeas, and white beans, as well as in peanuts and selected other botanical sources [10,11,12]. Hypaphorine has been detected in human milk in relation to maternal legume exposure [10], and a controlled crossover study identified urinary hypaphorine among endogenous or putative fermentation-derived metabolites discriminating acute consumption of lentils, chickpeas, and white beans [11]. A systematic review of legume-intake biomarkers therefore identified hypaphorine as a promising candidate requiring targeted confirmation in observational settings [12]. Whether targeted measurement of urinary hypaphorine can rank, discriminate, or quantitatively approximate habitual legume intake in free-living adults remains unclear.

A secondary question is whether a legume-related biomarker may also show clinically relevant correlates. Plant-rich dietary patterns have been associated with lower chronic low-grade inflammatory profiles [13], while leukocyte count is a routinely available indicator of systemic immune-inflammatory status [14]. Experimental studies provide additional, although indirect, biological plausibility. Hypaphorine has modulated stress-activated kinase and inflammatory signalling in preclinical models [15]. Such findings cannot establish effects in humans, but they justify cautious examination of clinical correlates in an exploratory framework.

The primary aim of the present study was to examine the association between urinary hypaphorine and the FFQ-derived estimate of legume intake within the EAT-Lancet food-group framework in free-living adults. Specifically, group-based and continuous associations were examined alongside the comparative association profile across prespecified plant-based dietary exposures and overall diet quality. Multivariable analyses tested independence from demographic, anthropometric, dietary, and urine-dilution covariates, and cross-validation assessed estimation and discrimination of the FFQ-derived legume-intake variable. A secondary exploratory aim was to examine clinical and biochemical correlates of urinary hypaphorine, with focused evaluation of the observed association with leukocyte count.

## 2. Materials and Methods

### 2.1. Study Design and Participants

This cross-sectional observational study was conducted within the Dietary Deal project (reference AC21/00038). The protocol was approved by the Ethics Committee of Hospital Clínico San Carlos, Madrid, Spain, on 7 June 2022 (protocol 22/363-E). Participants were recruited through the Endocrinology and Nutrition Department of Hospital Clínico San Carlos between November 2022 and June 2023; this was the recruitment setting, and eligibility was not restricted to patients with a single endocrine diagnosis. Recruitment was largely opportunistic and took place during routine outpatient visits. According to the original DIETARY DEAL substudy report, the recruitment pool included healthy hospital staff and patients attending the Endocrinology/Nutrition service. Patients with short-bowel syndrome or gastrointestinal-tract alterations and individuals taking oral supplements or vitamins were not included [8]. All participants provided written informed consent before enrolment. Participants attended a single study visit during which dietary, anthropometric, lifestyle, and clinical/biochemical data were collected [8,9,16].

The present analytical sample comprised 138 participants with matched dietary and urinary hypaphorine data; analyses involving leukocyte count used 137 complete cases. The cohort was clinically heterogeneous rather than restricted to a single endocrine diagnosis. In the structured medical-history data available for these 138 participants, dyslipidaemia was recorded in 33 participants, hypertension in 19, type 2 diabetes in 12, obesity in 10, and cardiovascular disease in 8. These characteristics were considered when assessing the generalisability of the exploratory clinical findings.

### 2.2. Anthropometric, Dietary, and Lifestyle Assessment

Habitual dietary intake was assessed by a trained dietitian using a validated semiquantitative FFQ comprising 136 food items [17]. Food intakes were converted to grams per day using reported consumption frequencies and standard portion sizes. The primary dietary exposure was the FFQ-derived continuous legume-intake variable, expressed in g/day and organised according to the EAT-Lancet food-group framework. This variable comprised the FFQ items for lentils, chickpeas, dry beans (pinto, white, or black beans), and peas/broad beans. Soy products were not assessed by the FFQ and therefore did not contribute to the variable. Daily legume intake was calculated by converting the reported frequency for each item to servings per day, summing these frequencies across the four legume items, and multiplying the resulting value by the standard cooked-legume portion of 60 g. Mixed dishes in which legumes constituted the principal food component, such as lentil-based dishes or chickpea salads, were recorded under the corresponding legume item and contributed to estimated legume intake using the same standard portion. Peanuts and peanut butter were assigned to the nuts group rather than the legume group. Accordingly, the continuous variable was expressed as cooked-weight intake. This continuous g/day variable was distinct from the scored EAT-Lancet legume component used to construct the overall index. For this component, legume intake was assigned 0–3 points according to the thresholds described by published thresholds [18]. (<18.75, 18.75–37.5, 37.5–75, and >75 g/day). No additional conversion to uncooked-weight equivalents was performed before assigning the 0–3-point legume-component score used in the overall EAT-Lancet index. The 14 component scores were summed to produce the 0–42-point EAT-Lancet score [18]. For comparative dietary analyses, a combined plant-food variable was defined as the sum of legumes, whole grains, vegetables, fruits, and nuts. Total energy intake was estimated from the FFQ. The median legume intake in the analytical sample (18.558 g/day) was used only for descriptive low/high comparisons. All principal association and prediction analyses retained legume intake as a continuous variable.

Body weight and height were measured using standardised procedures, and body mass index (BMI) was calculated as weight in kilograms divided by height in metres squared. Physical activity was recorded as total minutes per week using the International Physical Activity Questionnaire (IPAQ) [19].

### 2.3. Clinical and Biochemical Measurements

Fasting blood and urine samples were collected at the baseline visit. Routine clinical and biochemical measurements included glucose, insulin, glycated haemoglobin, lipid variables, serum folate, high-sensitivity C-reactive protein (hs-CRP), interleukin-6, liver enzymes, estimated glomerular filtration rate, blood-cell counts, and fibrinogen. HOMA-IR was calculated from fasting glucose and insulin. Following the initial descriptive and correlation analyses, leukocyte count (×10^3^/µL) was selected for focused exploratory modelling because it showed a consistent inverse pattern across both analyses. Clinical analyses used available-case data, and the focused leukocyte model included 137 participants. The broader clinical correlation screen was corrected using the Benjamini-Hochberg false-discovery rate (FDR).

### 2.4. Urinary Hypaphorine Quantification and Creatinine Signal

Urinary hypaphorine was quantified using a targeted metabolomics assay. First-morning urine samples were collected in sterile containers, aliquoted, and stored at −80 °C until analysis. Polar metabolites were extracted from 20 µL of urine using a biphasic procedure comprising LC–MS-grade methanol (VWR Chemicals, Barcelona, Spain), methyl tert-butyl ether (MTBE; Sigma-Aldrich, St. Louis, MO, USA), and ultrapure water obtained from a Millipore system (Billerica, MA, USA), as previously reported [16]. After phase separation, aliquots of the lower polar layer were evaporated to dryness and reconstituted in 110 µL of LC–MS-grade acetonitrile (ACN; VWR Chemicals, Barcelona, Spain) and ultrapure water (80:20, *v*/*v*) containing an internal-standard mixture comprising Val-Tyr-Val, 12-[[(cyclohexylamino)carbonyl]amino]-dodecanoic acid (CUDA), DL-alanine-d_3_, DL-glutamic acid-d_3_, choline-d_9_, ^1^^5^N_2_-L-arginine, L-methionine-d_8_, and L-phenylalanine-d_8_. Val-Tyr-Val was obtained from Sigma-Aldrich (St. Louis, MO, USA); CUDA from Cayman Chemical (Ann Arbor, MI, USA); DL-alanine-d_3_, DL-glutamic acid-d_3_, choline-d_9_, ^1^^5^N_2_-L-arginine, and L-methionine-d_8_ from Cambridge Isotope Laboratories, Inc. (Andover, MA, USA); and L-phenylalanine-d_8_ from LGC Standards (Toronto, ON, Canada). A 5-µL aliquot was analysed at the CIAL Metabolomics Platform using a Vanquish Horizon ultrahigh-performance liquid chromatography system coupled to an Orbitrap Exploris 120 mass spectrometer (both from Thermo Fisher Scientific, Bremen, Germany). Chromatographic separation was performed on an Acquity UPLC BEH Amide column (150 mm × 2.1 mm internal diameter; 1.7 µm particle size) fitted with an Acquity VanGuard BEH Amide pre-column (5 mm × 2.1 mm internal diameter; 1.7 µm particle size; both from Waters Corporation, Milford, MA, USA). Detection was performed using heated electrospray ionisation in positive mode. The hypaphorine analytical standard was obtained from BLD Pharmatech GmbH (Reinbek, Germany). Hypaphorine was quantified using external calibration curves analysed at the beginning, middle, and end of the injection sequence. Quantitative data were processed using Skyline software (version 24.1.0.199; MacCoss Lab, University of Washington, Seattle, WA, USA). Randomised injection order, pooled quality control (QC) samples, procedure blanks, and internal standards for retention-time correction were used for quality assurance. The linearity of the hypaphorine quantitative assay was initially evaluated using nine standard concentrations (0.0001, 0.0005, 0.001, 0.005, 0.01, 0.05, 0.1, 0.5, and 1 µg/mL) prepared in neat solvent [ACN:water (80:20, *v*/*v*)]. Sensitivity was assessed using the limit of detection (LOD), lower limit of quantification (LLOQ), and upper limit of quantification (ULOQ). The LOD was established as the concentration of the lowest calibration standard with a signal-to-noise ratio of at least 3, whereas a signal-to-noise ratio of at least 10 was required for the LLOQ. The ULOQ was determined by visual inspection of the calibration curves together with the coefficient of determination. Within-sequence precision was assessed from 16 pooled QC samples analysed throughout the sequence, approximately every 10 participant samples, and expressed as the relative standard deviation. Finally, the matrix effect (ME) was assessed by comparing the response of hypaphorine spiked at the same concentration in pooled QC urine and in neat solvent. Three aliquots of pooled QC urine were extracted as described above and reconstituted in 110 µL of ACN:water (80:20, *v*/*v*) spiked with hypaphorine at 0.01 µg/mL. In parallel, three additional pooled QC aliquots were extracted and reconstituted in the same solvent without added hypaphorine. All samples were analysed under the same analytical conditions, together with hypaphorine at 0.01 µg/mL in neat solvent. To account for endogenous hypaphorine in the pooled urine matrix, the peak area of the unspiked matrix was subtracted from that of the spiked matrix before calculation of the matrix effect using the following equation:

ME (%) = [(peak area in spiked pooled urine − peak area in unspiked pooled urine)/peak area in neat solvent] × 100

Laboratory validation documented a retention time of 5.55 min and a calibration coefficient of determination of 0.9997, with a quantitative range of 0.0001–0.1 µg/mL. The LOD and LLOQ were both 0.0001 µg/mL, and the ULOQ was 0.1 µg/mL. Within-sequence precision in pooled QC samples was 2.8%, and the ME was 68.5%. This ME value was applied as a common correction to hypaphorine peak areas in the diluted samples before back-calculation of concentrations in the original urine samples. Corrected hypaphorine peak areas from four participant samples exceeded the peak area of the experimental ULOQ point (0.1 µg/mL) and were therefore flagged as above the upper limit of quantification (>ULOQ). The ME was estimated using a pooled QC mixture comprising samples from the 138 participants and therefore represented a pooled-matrix estimate; because urine composition varies between individuals, participant-specific matrix effects may differ. Numerical extrapolations beyond the upper calibration point were available for the four >ULOQ samples. These observations were retained in the primary analyses and excluded in sensitivity analyses. Hypaphorine concentrations were log10-transformed for regression modelling because their distribution was right-skewed. Recovery and accuracy were not formally determined as separate validation parameters in this analytical workflow.

The urinary creatinine variable was obtained as a relative peak-height signal from a parallel untargeted urinary metabolomics dataset generated from the same first-morning urine samples. Absolute urinary creatinine was not measured in these samples. Urine specific gravity was also not measured. Serum creatinine was measured as part of the independent clinical biochemistry panel, but it is not a measure of urine concentration and was therefore not used for urine-dilution adjustment. For the untargeted urine analysis, polar metabolites were analysed with the instrumentation described above and processed using MS-DIAL software (version 4.9; RIKEN Center for Sustainable Resource Science, Yokohama, Japan), following the previously described peak-processing, alignment, and annotation workflow [16]. The resulting urinary creatinine signal was used as a prespecified covariate to account for between-person variation in urine concentration and should not be interpreted as an absolute clinical urinary creatinine measurement. The primary model included the log10 urinary creatinine signal as a covariate rather than relying solely on a metabolite-to-creatinine ratio. A ratio-based sensitivity analysis using the same relative creatinine signal was also performed.

### 2.5. Descriptive Comparisons and Correlation Analyses

Continuous variables are reported as mean ± standard deviation when approximately normally distributed and as median (interquartile range) otherwise. Categorical variables are presented as *n* (%). Low and high legume-intake groups were compared using Welch *t* tests, Mann-Whitney U tests, chi-square tests, or Fisher exact tests, as appropriate. Spearman rank correlations examined associations between urinary hypaphorine and a prespecified plant-based dietary family comprising legume intake, whole grains, vegetables, fruits, nuts, combined plant-food intake, and the EAT-Lancet score. Total energy intake was excluded from this correlation family because it was treated a priori as an adjustment covariate in multivariable analyses. Nominal *p* values were adjusted across the seven displayed dietary variables using the Benjamini-Hochberg procedure. Clinical and biochemical correlations were corrected separately across the 24 variables included in the clinical screening family using the Benjamini-Hochberg procedure.

### 2.6. Multivariable Modelling and Influence Analyses

The primary multivariable model used log10 urinary hypaphorine as the dependent variable and continuous legume intake as the predictor of interest, adjusting a priori for age, sex, BMI, total energy intake, and the log10 urinary creatinine signal. Ordinary least-squares coefficients were accompanied by HC3 heteroscedasticity-consistent standard errors and 95% confidence intervals. The legume coefficient was back-transformed to express the percentage difference in hypaphorine associated with each 10 g/day increment in legume intake. Sensitivity analyses excluded >ULOQ observations and modelled a creatinine-normalised hypaphorine ratio.

A secondary model added leukocyte count to the hypaphorine model. The reciprocal exploratory clinical model used leukocyte count as the dependent variable and included log10 hypaphorine, legume intake, age, sex, BMI, total energy intake, and the log10 urinary creatinine signal. For comparison, an analogous exploratory model was fitted with triglyceride concentration as the dependent variable using the same predictor set and HC3 inference. Variance-inflation factors were examined to assess collinearity. Model diagnostics comprised residual and influence measures, including Cook’s distance, leverage, and DFFITS. Sensitivity analyses of the leukocyte model evaluated exclusions based on influential observations, standardised residuals, and Cook’s distance; robust regression with case-bootstrap inference; and exclusion of >ULOQ observations. For graphical presentation of the primary hypaphorine and leukocyte models, the outcome and continuous predictors were standardised to z-scores, whereas sex was retained as a binary variable. Additional clinically motivated sensitivity analyses further adjusted the leukocyte model for smoking status (non-smoker, former smoker, current smoker) or FFQ-derived alcohol intake (g/day), replaced BMI with waist circumference (cm) to assess central adiposity, and repeated the primary leukocyte model after excluding participants with clearly identifiable chronic inflammatory or autoimmune conditions recorded in the medical history. Acute infection at the sampling visit and corticosteroid exposure could not be reliably ascertained and were not modelled.

### 2.7. Cross-Validated Evaluation of Quantitative Estimation and Discrimination

To assess whether hypaphorine could help estimate the questionnaire-derived exposure, rather than merely show an association with it, the FFQ-derived legume-intake variable (g/day) was modelled as the outcome. Four prespecified models were compared. These comprised an intercept-only benchmark, a covariate model containing age, sex, BMI, and energy intake, a biomarker model containing log10 hypaphorine and the log10 urinary creatinine signal, and a combined model containing biomarker and covariate terms. Performance was evaluated using 100 repetitions of 10-fold cross-validation. Quantitative metrics were root-mean-square error (RMSE), mean absolute error (MAE), cross-validated R^2^, Spearman correlation between observed and predicted values, and calibration slope.

As a secondary classification task, logistic models distinguished participants with intake above the sample median from those with intake at or below it. Performance was summarised using the area under the receiver-operating-characteristic curve (AUC), Brier score, and balanced accuracy. All reported performance estimates were obtained by cross-validation against the FFQ-derived legume-intake variable and were interpreted according to transparent prediction-model reporting principles [20]. Cross-validated performance was also re-evaluated after excluding >ULOQ observations. These analyses evaluated agreement with the FFQ-derived legume-intake variable and did not constitute validation against objectively measured true intake.

### 2.8. Exploratory Mediation Analysis

An exploratory cross-sectional mediation model examined whether log10 urinary hypaphorine statistically accounted for part of the association between legume intake and leukocyte count. Legume intake was scaled per 10 g/day. The mediator and outcome models were adjusted for age, sex, BMI, total energy intake, and the log10 urinary creatinine signal. The indirect effect was calculated as the product of the a path (legume intake to hypaphorine) and b path (hypaphorine to leukocytes conditional on legume intake). Percentile 95% confidence intervals and two-sided empirical *p* values were obtained from 5000 nonparametric case-bootstrap resamples. Because exposure, mediator, and outcome were measured cross-sectionally, the model was interpreted solely as a hypothesis-generating statistical decomposition and not as evidence of temporal or causal mediation [21,22].

### 2.9. Statistical Software and Significance Thresholds

Analyses were performed in R version 4.4.3 (R Foundation for Statistical Computing, Vienna, Austria) using two-sided tests. Nominal *p* < 0.05 was considered statistically significant for the prespecified primary dietary model. Plant-based dietary and clinical screening results were interpreted using both nominal *p* values and BH-FDR *q* values, with FDR control applied separately within each family. Focused leukocyte analyses were treated as exploratory because the outcome was prioritised after the descriptive and correlation analyses. Influence-based exclusions were used only in sensitivity analyses.

## 3. Results

### 3.1. Participant Characteristics by FFQ-Derived Legume Intake

The analytical sample included 138 adults, of whom 96 (69.6%) were women. The median FFQ-derived legume intake was 18.558 g/day, yielding 69 participants in each descriptive intake group. Median intake was 12.56 (7.99–15.98) g/day in the low group and 29.70 (25.13–37.70) g/day in the high group (*p* < 0.001). Age, sex, BMI, and physical activity did not differ materially between groups (Table 1). The urinary creatinine signal, used as a urine-dilution covariate, was also similar in the low- and high-legume groups (1.37 ± 0.27 vs. 1.42 ± 0.27 × 10^9^ peak height, respectively; *p* = 0.200). Participants in the high-legume group also reported greater whole-grain, vegetable, fruit, and nut intake and had a higher EAT-Lancet score. This clustering provided the dietary-pattern context for the continuous and multivariable analyses.

Among clinical variables, leukocyte count was lower in the high-legume group [5.45 (4.70–6.50) vs. 6.00 (5.40–7.40) × 10^3^/µL; *p* = 0.0026] (Table 2). Apart from leukocyte count, no other clinical or biochemical variable differed significantly between intake groups.

### 3.2. Urinary Hypaphorine and Dietary Association Profile

**Figure 1:**
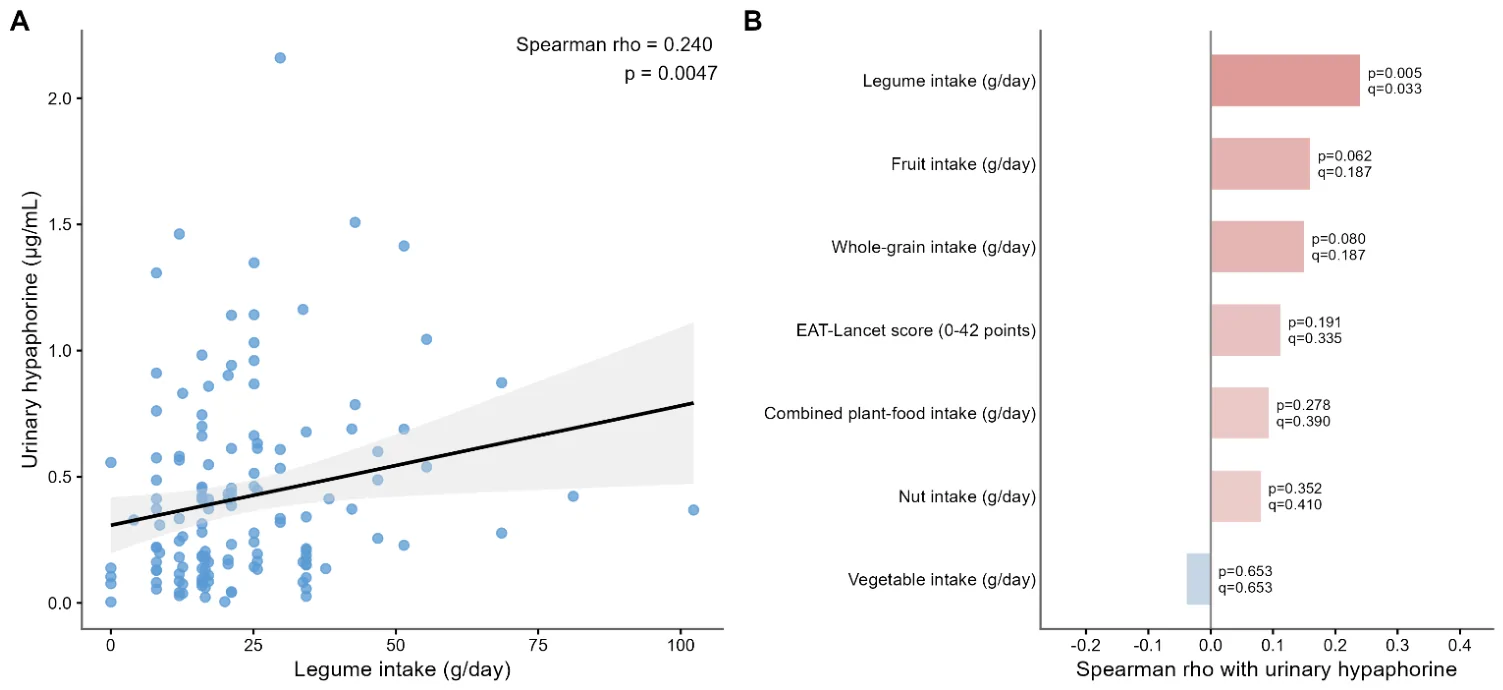
Plant-based dietary associations of urinary hypaphorine. (**A**) Continuous association between urinary hypaphorine concentration and the FFQ-derived legume-intake variable. Points represent individual participants. The line and 95% confidence band provide a visual summary of the association. (**B**) Spearman correlation profile across the prespecified plant-based dietary family comprising legume intake, fruit intake, whole-grain intake, the EAT-Lancet score, combined plant-food intake, nut intake, and vegetable intake. Combined plant-food intake was calculated as the sum of legume, whole-grain, vegetable, fruit, and nut intakes, expressed in g/day. Nominal *p* values and Benjamini-Hochberg FDR-adjusted *q* values are displayed.

Urinary hypaphorine was higher in the high-legume group than in the low-legume group [0.41 (0.19–0.68) vs. 0.20 (0.10–0.45) µg/mL; *p* = 0.0025; Table 1]. The association remained after excluding the four >ULOQ observations (*p* = 0.0043). Across the full intake range, hypaphorine correlated positively with legume intake (*ρ* = 0.240; *p* = 0.0047; Figure 1A); the result remained after excluding >ULOQ observations (*ρ* = 0.219; *p* = 0.0109).

Within the prespecified plant-based dietary family (Figure 1B), legume intake showed the largest observed association with urinary hypaphorine (*ρ* = 0.240; *p* = 0.005; *q* = 0.033). No other displayed plant-based exposure remained significant after BH-FDR correction, including fruits (*ρ* = 0.160; *p* = 0.062; *q* = 0.187), whole grains (*ρ* = 0.149; *p* = 0.080; *q* = 0.187), the EAT-Lancet score (*ρ* = 0.112; *p* = 0.191; *q* = 0.335), combined plant-food intake (*ρ* = 0.093; *p* = 0.278; *q* = 0.390), nuts (*ρ* = 0.080; *p* = 0.352; *q* = 0.410), or vegetables (*ρ* = −0.039; *p* = 0.653; *q* = 0.653). These results indicate that the dietary association was most evident for legumes, but do not establish food specificity because hypaphorine occurs in other botanical sources and correlated dietary behaviours may contribute. Complete dietary correlation results, including the sensitivity analysis excluding >ULOQ observations, are reported in Table S1.

### 3.3. Adjusted Continuous Association with Urinary Hypaphorine

**Figure 2:**
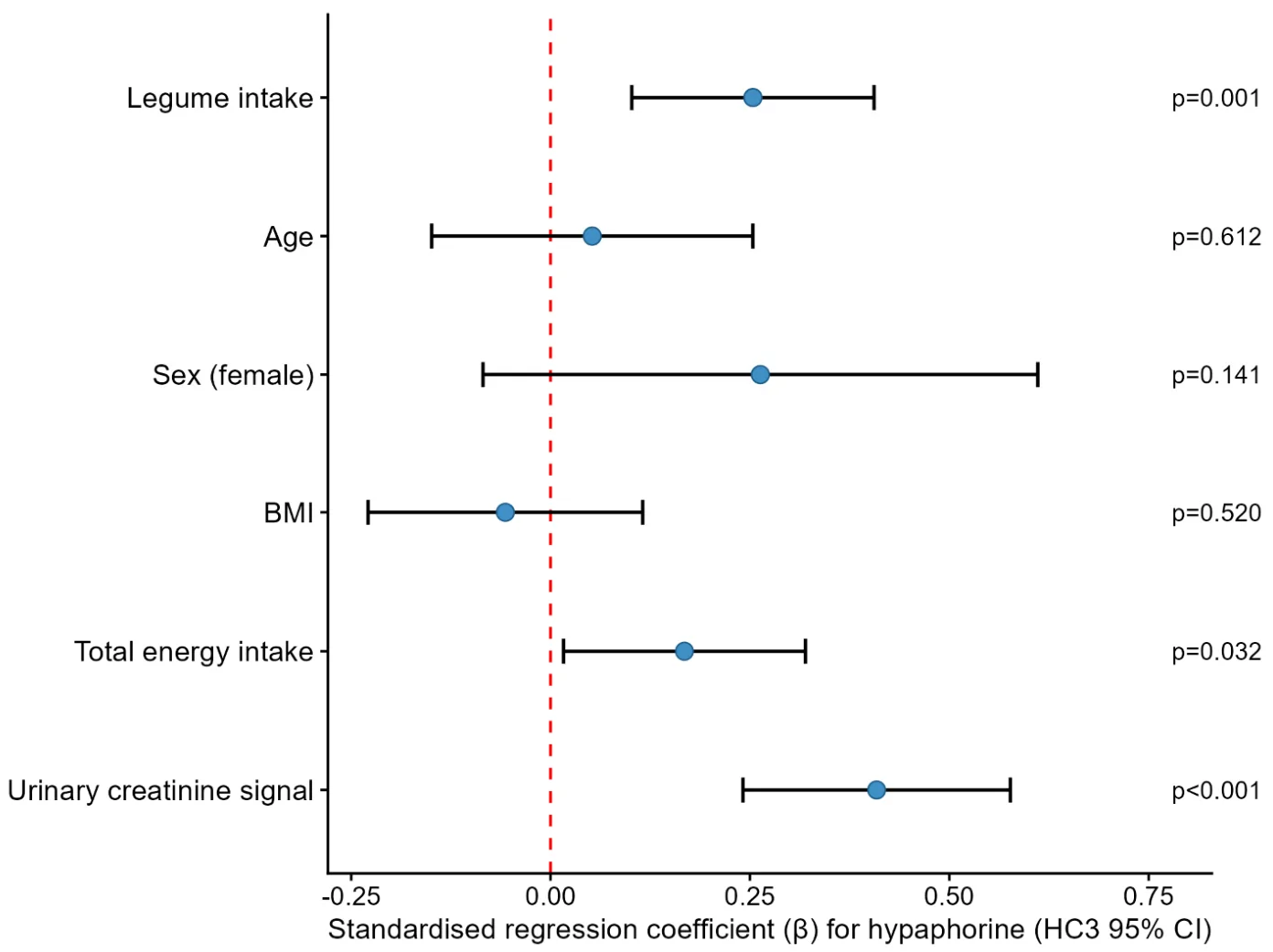
Multivariable model with log10 urinary hypaphorine as the outcome. Standardised regression coefficients and HC3 95% confidence intervals are shown for the FFQ-derived legume-intake variable, age, sex, BMI, total energy intake, and the log10 urinary creatinine signal. The outcome and continuous predictors were standardised before modelling. Coefficients for continuous predictors therefore represent the difference in the standardised outcome per one-standard-deviation increase in the predictor. The coefficient for sex represents the adjusted difference between female and male participants. Unstandardised coefficients in the original measurement scales are reported in Table S2.

In the prespecified multivariable model, legume intake remained positively associated with log10 urinary hypaphorine after adjustment for age, sex, BMI, total energy intake, and the log10 urinary creatinine signal (*β* = 0.00757 per g/day; 95% CI, 0.00304–0.01211; *p* = 0.0014; Figure 2 and Table S2). This corresponds to 19.1% higher urinary hypaphorine for each 10 g/day increment in legume intake (95% CI, 7.2–32.2%). The urinary creatinine signal was included as the prespecified urine-dilution covariate. The model had an *R*^2^ of 0.252 and an adjusted *R*^2^ of 0.217.

The legume coefficient remained significant after exclusion of >ULOQ observations (*β* = 0.00701 per g/day; *p* = 0.0020; approximately 17.5% higher hypaphorine per 10 g/day) and in the creatinine-ratio sensitivity model (*β* = 0.00721; *p* = 0.0005; approximately 18.1% higher per 10 g/day). Adding leukocyte count modestly increased model fit and did not materially attenuate the legume association (*β* = 0.00710; *p* = 0.0010). Variance-inflation factors were low (maximum approximately 1.5).

### 3.4. Cross-Validated Performance for Estimating and Discriminating FFQ-Derived Legume Intake

Cross-validation tempered the interpretation of hypaphorine as a quantitative intake predictor. The biomarker model produced the best overall quantitative performance for the FFQ-derived legume-intake variable, with mean cross-validated RMSE of 15.19 g/day, MAE of 10.81 g/day, cross-validated R^2^ of 0.061, and a Spearman correlation between observed and predicted values of 0.305 (Table 3). Compared with the intercept-only benchmark, the biomarker model reduced RMSE by 3.8% and MAE by 5.6%. The combined model did not improve prediction error or cross-validated R^2^ relative to the biomarker model (RMSE, 15.26 g/day; cross-validated R^2^, 0.052), indicating that age, sex, BMI, and energy intake added little out-of-sample information.

Predictions were compressed toward the cohort mean, as reflected by a biomarker-model calibration slope of 0.80 and by systematic overestimation of low intakes and underestimation of high intakes. For discrimination above versus at or below the median, the biomarker model achieved AUC = 0.653 and balanced accuracy = 0.619. Performance remained similar after excluding >ULOQ observations (AUC = 0.646). These findings indicate modest cross-validated ranking and discrimination of the FFQ-derived legume-intake variable rather than precise recovery of individual consumption in g/day.

### 3.5. Exploratory Clinical Correlation Profile and Focused Leukocyte Analysis

**Figure 3:**
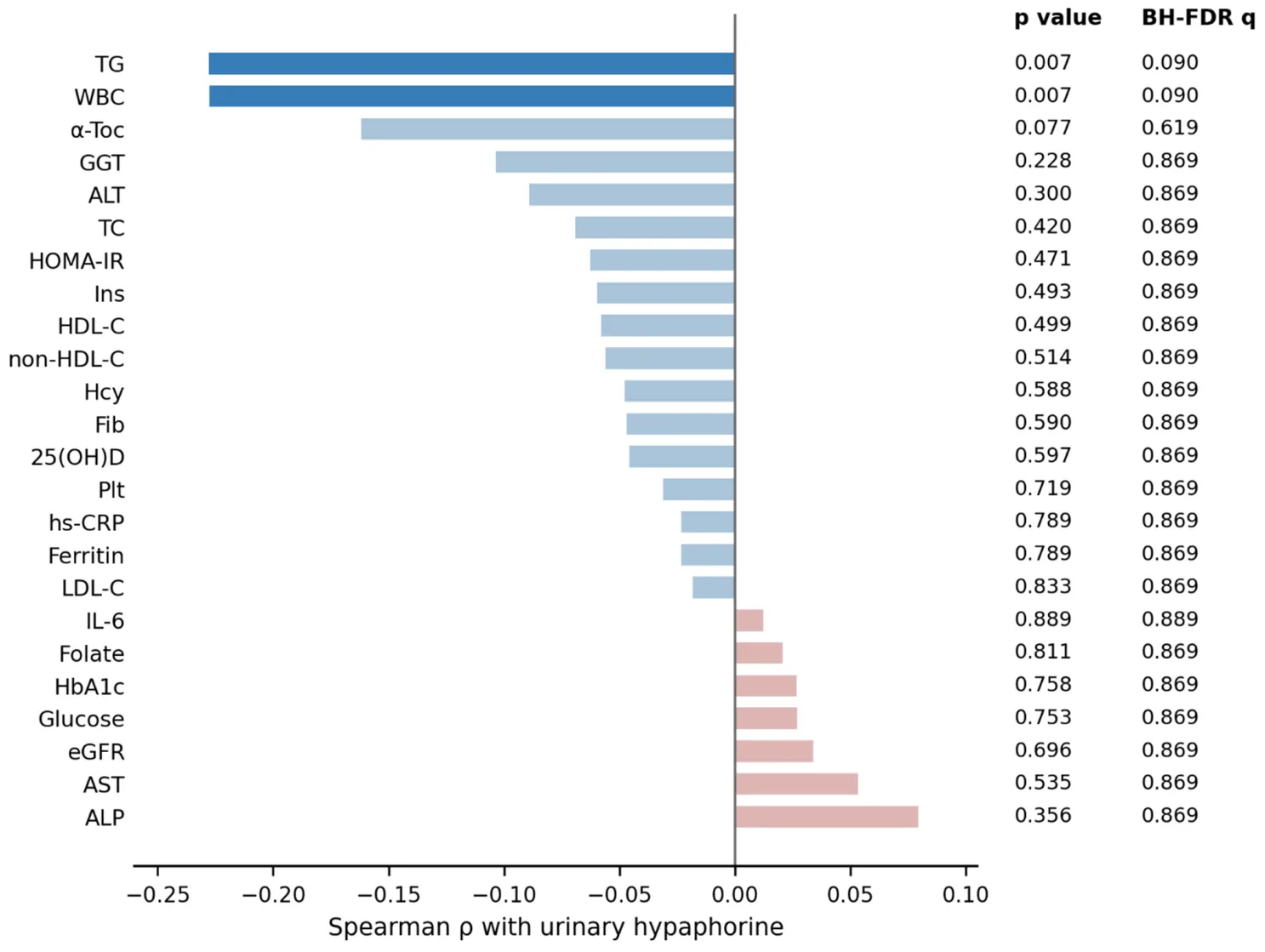
Clinical and biochemical correlation profile of urinary hypaphorine. Spearman correlations are ordered from the most negative to the most positive association. Nominal *p* values and Benjamini-Hochberg FDR-adjusted *q* values are displayed in aligned columns. Abbreviations: 25(OH)D, 25-hydroxyvitamin D; ALP, alkaline phosphatase; ALT, alanine aminotransferase; AST, aspartate aminotransferase; eGFR, estimated glomerular filtration rate; Fib, fibrinogen; GGT, gamma-glutamyltransferase; HbA1c, glycated haemoglobin; Hcy, homocysteine; HDL-C, high-density lipoprotein cholesterol; HOMA-IR, homeostatic model assessment of insulin resistance; hs-CRP, high-sensitivity C-reactive protein; IL-6, interleukin-6; Ins, insulin; LDL-C, low-density lipoprotein cholesterol; non-HDL-C, non-high-density lipoprotein cholesterol; Plt, platelets; TC, total cholesterol; TG, triglycerides; WBC, white blood cell count; α-Toc, α-tocopherol.

In the exploratory clinical screen (Figure 3), leukocytes (*ρ* = −0.228; *p* = 0.0075; *q* = 0.090) and triglycerides (*ρ* = −0.228; *p* = 0.0074; *q* = 0.090) showed inverse nominal associations that did not remain below the 0.05 FDR threshold. No other nominally significant correlations were observed between hypaphorine and hs-CRP, insulin, HOMA-IR, glucose, interleukin-6, serum folate, or the remaining measured clinical variables. Complete clinical and biochemical correlation results, including the sensitivity analysis excluding >ULOQ observations, are reported in Table S3. An analogous adjusted model with triglycerides as the outcome did not support an independent association with hypaphorine (*p* = 0.424), whereas the leukocyte coefficient remained nominally significant in the focused exploratory model.

**Figure 4:**
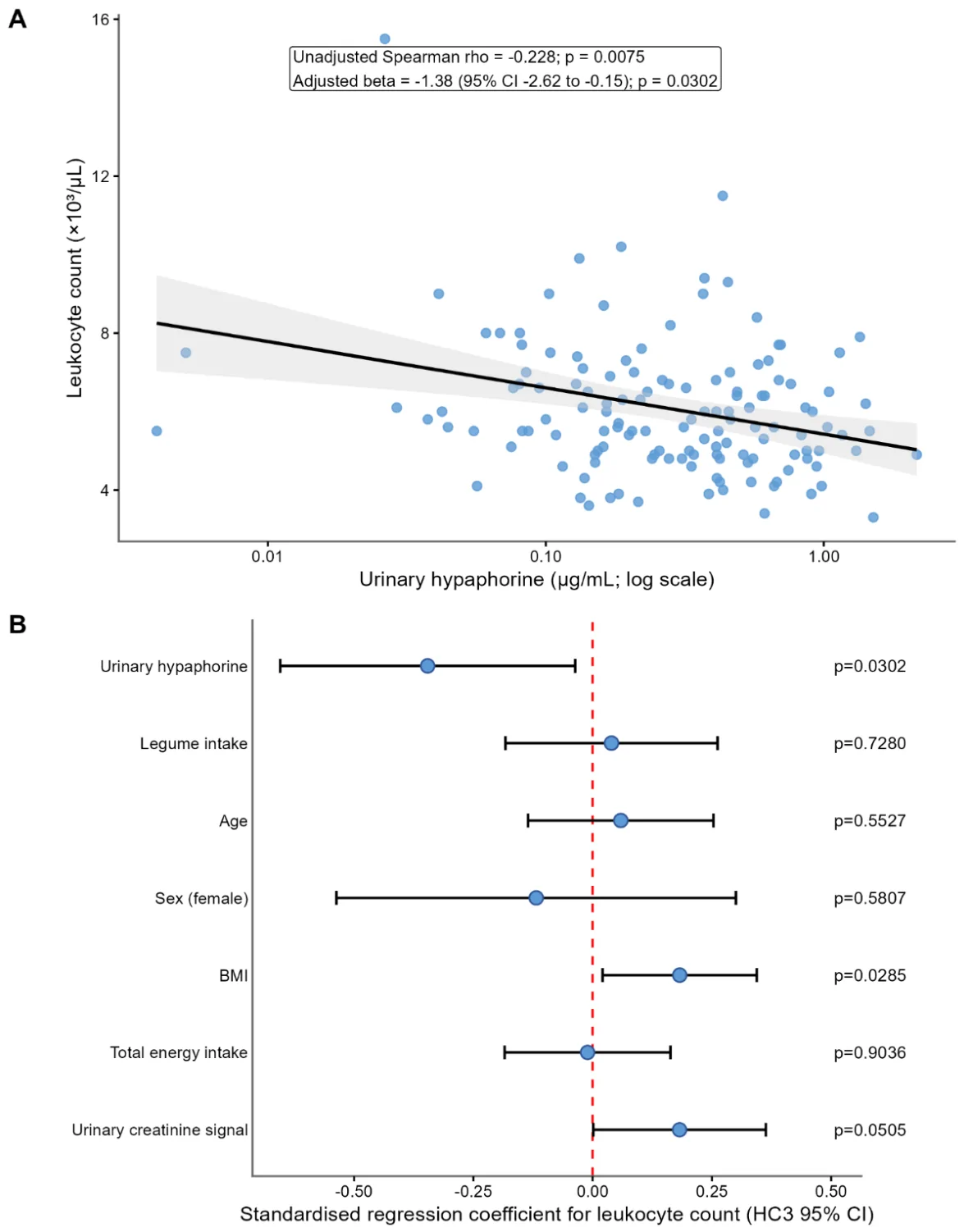
Exploratory leukocyte analyses in relation to urinary hypaphorine. (**A**) Continuous association between urinary hypaphorine concentration and leukocyte count. Points represent individual participants. The fitted line and 95% confidence band provide a visual summary of the unadjusted association. The annotation reports the unadjusted Spearman correlation and the adjusted unstandardised hypaphorine coefficient from the model shown in panel (**B**). (**B**) Multivariable model with leukocyte count as the outcome. Standardised regression coefficients and HC3 95% confidence intervals are shown for log10 urinary hypaphorine, the FFQ-derived legume-intake variable, age, sex, BMI, total energy intake, and the log10 urinary creatinine signal. The outcome and continuous predictors were standardised before modelling. The coefficient for sex represents the adjusted difference between female and male participants. Unstandardised coefficients in the original measurement scales are reported in Table S2.

The unadjusted continuous association between urinary hypaphorine and leukocyte count is shown in Figure 4A. In the post-screening focused exploratory leukocyte model, each tenfold higher urinary hypaphorine concentration was associated with a 1.38 × 10^3^/µL lower leukocyte count (*β* = −1.382; 95% CI, −2.618 to −0.146; HC3 *p* = 0.030; Figure 4B and Table S2). Legume intake was not independently associated with leukocyte count after inclusion of hypaphorine (*p* = 0.728). BMI showed a modest positive association with leukocytes (*p* = 0.029). The model had an *R*^2^ of 0.157 and an adjusted *R*^2^ of 0.112.

### 3.6. Robustness of the Leukocyte Association

The inverse direction of the hypaphorine-leukocyte coefficient was retained in every planned sensitivity analysis. Nominal statistical support remained after excluding both extreme-residual observations, after excluding observations with Cook’s distance > 4/*n*, after excluding >ULOQ observations, and in Huber-weighted robust regression. The robust-regression estimate was −0.810 (bootstrap 95% CI, −1.563 to −0.213; *p* = 0.011). Exclusion of the single most influential observation attenuated the association (*p* = 0.061) without reversing its direction. The coefficient remained negative across all sensitivity analyses, although its precision varied. The full sensitivity profile is presented in Figure S1 and Table S4. Additional clinically motivated analyses yielded similar hypaphorine coefficients after adjustment for smoking status (β = −1.38, 95% CI −2.61 to −0.15; *p* = 0.030) or alcohol intake (β = −1.39, 95% CI −2.64 to −0.14; *p* = 0.031), replacement of BMI with waist circumference (β = −1.32, 95% CI −2.55 to −0.08; *p* = 0.039), and exclusion of 10 participants with recorded chronic inflammatory or autoimmune conditions (β = −1.36, 95% CI −2.66 to −0.07; *p* = 0.042; Table S4).

### 3.7. Exploratory Indirect Association Through Hypaphorine

An exploratory cross-sectional mediation analysis yielded an estimated indirect effect of −0.1036 × 10^3^/µL per 10-g/day greater legume intake (bootstrap 95% CI, −0.2123 to −0.0233; *p* = 0.0044), whereas the total effect of legume intake on leukocyte count was not significant (*p* = 0.5536). Because legume intake, urinary hypaphorine, and leukocyte count were measured cross-sectionally, this statistical decomposition does not establish temporal ordering or biological mediation and is presented only as hypothesis-generating. Full path estimates are reported in Figure S2 and Table S5.

## 4. Discussion

Targeted urinary hypaphorine was positively associated with the FFQ-derived estimate of legume intake within the EAT-Lancet food-group framework in 138 free-living adults, and the association remained after adjustment for demographic, dietary, and urine-dilution covariates. Concentrations were higher among participants in the higher-intake group, and each additional 10 g/day of estimated legume intake was associated with approximately 19% higher urinary hypaphorine. The association was robust in analyses excluding >ULOQ observations and using creatinine normalisation. Legume intake showed the largest observed correlation within the prespecified dietary family. Together, these findings support urinary hypaphorine as a candidate marker of relative exposure to the FFQ-derived legume-intake variable, while its limited quantitative performance precludes interpretation as a measure of true individual consumption. The inverse association with leukocyte count adds an exploratory clinical hypothesis rather than evidence of a health effect.

Previous controlled studies established the biological plausibility of hypaphorine as a legume-related metabolite. Keller et al. detected hypaphorine in human milk in relation to dietary exposure [10], and Garcia-Aloy et al. identified urinary hypaphorine among metabolites that discriminated lentil, chickpea, and white-bean meals [11]. The current study extends that evidence by quantifying urinary hypaphorine using targeted LC-MS with external calibration and documented assessment of within-sequence precision and matrix effects. It also demonstrates an adjusted association across the FFQ-derived legume-intake range and directly evaluates cross-validated performance. These are distinct stages of biomarker development, and their combination provides a stronger basis for practical interpretation than association testing alone [23,24].

The stronger relation with legumes than with combined plant-food intake, EAT-Lancet score, vegetables, or nuts is particularly informative because higher legume consumers also reported greater intake of several other plant foods. Such clustering is expected within healthy and sustainable dietary patterns [3,25] and can make it difficult to distinguish a food-specific signal from a general marker of diet quality. Previous Dietary Deal analyses have shown that urinary and nutrimetabolic information can classify broader dietary patterns and selected food-group exposures, while also illustrating how urinary metabolites may reflect either direct dietary exposure or more integrated diet-metabolism relationships [8,9,19]. Here, the targeted analysis narrows that framework to a chemically plausible legume marker. Hypaphorine is nevertheless present in peanuts and other botanical sources, and weak nominal trends with fruits and whole grains may partly reflect correlated plant-food behaviours. The current data therefore support relative enrichment of the signal for legume exposure rather than absolute food specificity, in agreement with recent reviews of urinary dietary biomarkers [26].

Association alone does not establish biomarker validity or practical utility. Validation frameworks distinguish candidate discovery from confirmation, dose-response assessment, robustness, analytical performance, and external reproducibility [23,26]. These frameworks also recognise that many urinary food biomarkers may be more useful for ranking or discriminating exposure than for recovering the exact amount consumed, particularly when a spot urine sample is compared with a questionnaire-derived estimate intended to reflect habitual intake [6,23].

The cross-validated prediction analysis adds an important practical layer to the biomarker assessment. The biomarker model showed small improvements over the intercept-only benchmark for estimating the FFQ-derived legume-intake variable, but overall quantitative performance remained limited. Cross-validated R^2^ was 0.061, and individual predictions were compressed toward the cohort mean. The AUC of approximately 0.65 and cross-validated rank correlation near 0.30 indicate modest cross-validated ranking information rather than accurate reconstruction of intake in g/day. A metabolite measured in a single spot-urine sample may therefore provide objective information about relative intake while remaining insufficient to replace detailed dietary assessment. Controlled feeding studies can achieve stronger classification under standardised conditions [7], whereas free-living performance is expected to be lower because timing, food combinations, and habitual behaviour are less controlled [24]. Quantitative calibration ultimately requires known intake, repeated reference measurements, and external validation [6,23].

The modest quantitative performance may partly reflect the mismatch between the biological and dietary-assessment windows. A spot urinary concentration reflects relatively recent exposure and is influenced by absorption, host and microbial metabolism, renal handling, and hydration, whereas the FFQ-derived legume-intake variable estimates longer-term average intake and is affected by recall and portion-size error. Repeated urine collections, time-stamped food records, and controlled dose-response studies should reduce this mismatch. Pulse-intervention studies have identified hypaphorine together with pipecolic-acid derivatives and other candidate metabolites [11,12], suggesting that complementary panels may capture pulse species, preparation methods, and interindividual metabolic variation more effectively than a single compound. Such panels would be especially valuable if evaluated with prespecified algorithms and independent validation samples [20,24].

Participants with higher legume intake had lower leukocyte counts; however, the absolute difference between group medians was 0.55 × 10^3^/µL, and the present study was not designed to determine whether a difference of this magnitude is clinically meaningful. This secondary observation is compatible with meta-analytic and trial evidence suggesting that non-soy legume consumption may improve selected inflammatory profiles, particularly in overweight or metabolically impaired populations [27,28]. Legume-enriched interventions have also reported reductions in inflammatory markers in overweight and diabetic adults [29,30]. In the present cohort, however, hypaphorine was not associated with hs-CRP, insulin, HOMA-IR, or most other clinical variables, indicating that it should not be portrayed as a broad marker of cardiometabolic health.

Population evidence further suggests that greater vegetable intake may be associated with lower white blood cell counts [31]. These observations provide a rationale for exploring leukocyte count alongside dietary-biomarker evaluation, without implying causality. Leukocyte count emerged from the descriptive and correlation analyses as a secondary signal requiring independent confirmation. The screening correlation did not remain below the 0.05 FDR threshold (*q* = 0.090), and exclusion of the single most influential observation attenuated the adjusted hypaphorine coefficient to *p* = 0.061. Higher leukocyte counts are widely used as an accessible indicator of low-grade systemic inflammation and adverse cardiometabolic prognosis [14]. Although the inverse hypaphorine-leukocyte coefficient remained directionally stable across the remaining sensitivity analyses, the variability in statistical support reinforces the exploratory nature of the finding. The analogous adjusted triglyceride model did not support an independent association. These patterns support a focused hypothesis for follow-up rather than a confirmatory clinical conclusion. The coefficient was also materially unchanged in sensitivity analyses addressing smoking status, alcohol intake, central adiposity, and recorded chronic inflammatory or autoimmune conditions, suggesting that none of these measured factors individually explained the observed association.

An additional, although still preliminary, link concerns the gut microbiota. In a recent mouse study, *Bifidobacterium adolescentis* administration increased hypaphorine abundance, while hypaphorine treatment attenuated acetaminophen-induced liver injury through hepatic Cry1 signalling [32]. However, the microbial pathway was not established, and de novo bacterial synthesis of hypaphorine was not demonstrated. Together with the anti-inflammatory effects reported in LPS-stimulated macrophages through inhibition of NF-κB and ERK signalling [33], these findings position hypaphorine as a potentially microbiota-associated, diet-derived metabolite at the food-microbiota-host interface, rather than as a confirmed postbiotic. Because gut-microbiota composition and function were not assessed in the present study, this interpretation remains hypothetical. A separate and more general microbiota-related pathway may arise from the microbial fermentation of legume-derived dietary fibre to short-chain fatty acids (SCFAs) [34]. These microbial metabolites can regulate lymphocyte and innate immune-cell functions through G-protein-coupled receptor signalling and histone-deacetylase inhibition [35]. This pathway may provide a broader biological context for the observed hypaphorine-leukocyte association, although its specific contribution remains to be established. Future studies should therefore combine controlled legume exposure with repeated urinary hypaphorine measurements, microbiome profiling, SCFA quantification, and immune-related outcomes.

The supplementary mediation analysis provided an exploratory statistical decomposition of the observed associations. Given the cross-sectional design, absence of temporal ordering, and non-significant total effect, it cannot support biological mediation and is retained solely as a hypothesis-generating analysis.

Several features support the main dietary-biomarker finding. Hypaphorine was quantified using a targeted LC-MS assay with external calibration and documented analytical precision and matrix effects. Urine dilution was addressed using a creatinine peak-height signal from the separate untargeted metabolomics dataset, with a ratio-based approach examined as a sensitivity analysis. Covariates were prespecified, and heteroscedasticity-consistent inference was used. Values above the ULOQ and influential observations were assessed in sensitivity analyses, and predictive performance was evaluated through repeated cross-validation. The analyses distinguished the primary dietary objective from the exploratory clinical findings.

The main limitations concern design and reference measurement. Cross-sectional data cannot establish temporality or causality, a single spot-urine sample may not represent usual excretion, and the interval between recent legume intake and sampling was not standardised. The FFQ-derived legume-intake variable is not a criterion measure of true consumption. Sample size was moderate, and cross-validation cannot replace testing in an independent population. Neither quantitative urinary creatinine nor urine specific gravity was available; urine dilution was therefore addressed using a relative untargeted-metabolomics creatinine peak-height signal, and residual dilution-related variability may remain. The matrix-effect correction was derived from pooled urine and may not fully capture participant-specific differences in urinary matrix composition. Hypaphorine is not exclusive to legumes, pulse subtypes were not modelled, and the clinical screen involved multiple outcomes. The leukocyte screening association did not remain below the 0.05 FDR threshold, and exclusion of the most influential observation attenuated the focused adjusted association to *p* = 0.061. The leukocyte analysis is therefore exploratory and requires independent replication. Additional sensitivity analyses addressing smoking status, alcohol intake, central adiposity, and recorded chronic inflammatory or autoimmune conditions produced similar estimates. However, acute infection at the sampling visit and corticosteroid exposure could not be reliably ascertained from the available dataset and remain potential sources of residual confounding. Nevertheless, the consistency of the primary adjusted dietary association, targeted quantification, and modest cross-validated ranking performance provide a basis for external validation. Future studies should combine controlled dose-response feeding, repeated timed or 24-h urine collections, detailed pulse-subtype records, and absolute urinary creatinine measurement. They should also test whether within-person changes in legume intake are accompanied by corresponding shifts in hypaphorine and whether these responses precede alterations in leukocyte counts or inflammatory phenotypes. The single-centre recruitment strategy, which included healthy hospital staff and patients seen through an Endocrinology/Nutrition service rather than a population-based sample, may also limit generalisability.

## 5. Conclusions

Urinary hypaphorine was positively associated with the FFQ-derived estimate of legume intake within the EAT-Lancet food-group framework and provided modest information for ranking participants according to this FFQ-derived exposure. Its limited quantitative performance and the use of a single urine sample indicate that it is not yet a stand-alone quantitative biomarker of habitual individual intake. The inverse association with leukocyte count was exploratory, of uncertain clinical relevance, and requires independent confirmation.

## Figures and Tables

**Table 1:** Participant, dietary, and urinary hypaphorine characteristics according to FFQ-derived legume intake.

Variable	Low Legume Intake (*n* = 69)	High Legume Intake (*n* = 69)	*p* Value
Urinary hypaphorine (µg/mL)	0.20 (0.10–0.45)	0.41 (0.19–0.68)	**0.0025**
Female sex, *n* (%)	48 (69.6%)	48 (69.6%)	1.0000
Age (years)	41 (28–53)	44 (31–56)	0.5091
BMI (kg/m^2^)	25.6 (22.0–31.6)	25.4 (21.9–29.6)	0.5726
Physical activity (min/week)	465 (315–840)	600 (420–855)	0.5561
Legume intake (g/day)	12.6 (8.0–16.0)	29.7 (25.1–37.7)	**<0.001**
Whole-grain intake (g/day)	5.3 (0.0–62.9)	34.3 (0.0–80.0)	**0.0140**
Potato intake (g/day)	31.4 (20.0–64.3)	42.8 (20.0–74.3)	0.1547
Vegetable intake (g/day)	400 (305–671)	598 (414–831)	**0.0020**
Fruit intake (g/day)	178 (107–375)	305 (164–421)	**0.0094**
Dairy intake (g/day)	360 (220–564)	346 (230–555)	0.6517
Beef/lamb intake (g/day)	21.4 (10.0–64.3)	21.4 (10.0–64.3)	0.8794
Pork intake (g/day)	78.1 (52.4–117.1)	63.8 (39.0–99.0)	0.1033
Poultry intake (g/day)	64.3 (42.8–74.3)	64.3 (21.4–74.3)	0.4716
Egg intake (g/day)	25.7 (25.7–25.7)	25.7 (25.7–47.1)	0.0624
Fish intake (g/day)	81.3 (58.1–116.3)	81.6 (54.7–117.0)	0.9135
Nut intake (g/day)	21.4 (3.3–21.4)	21.4 (7.1–50.0)	**0.0216**
Unsaturated oil intake (g/day)	66.7 (47.1–132.1)	66.7 (49.5–132.8)	0.6194
Added sugar intake (g/day)	0.67 (0.00–10.0)	2.09 (0.00–10.0)	0.4057
Total energy intake (kcal/day)	2128 (1833–2553)	2328 (1945–2661)	0.0730
EAT-Lancet score (0–42 points)	23.0 (21.0–25.0)	25.0 (23.0–27.0)	**<0.001**

Values are median (IQR), mean ± SD, or *n* (%), as indicated. Participants were classified according to the median legume intake of 18.558 g/day: low intake, <18.558 g/day; high intake, >18.558 g/day. No participant had an intake equal to the median. Statistically significant *p* values are shown in bold.

**Table 2:** Clinical and biochemical characteristics according to FFQ-derived legume intake.

Variable	Low Legume Intake	*n*	High Legume Intake	*n*	*p* Value
Glucose (mg/dL)	93.0 (85.0–101.0)	69	92.0 (87.0–98.0)	69	0.5380
Insulin (µIU/mL)	8.80 (4.75–15.6)	68	6.75 (4.08–12.4)	66	0.0873
Haemoglobin A1c (%)	5.40 (5.20–5.70)	69	5.40 (5.25–5.65)	67	0.7134
HOMA-IR	2.05 (1.10–3.91)	68	1.59 (0.87–2.79)	66	0.0947
Triglycerides (mg/dL)	81.5 (59.8–122.5)	68	74.0 (57.0–99.0)	69	0.1346
Total cholesterol (mg/dL)	183 (162–218)	69	186 (162–212)	69	0.9338
HDL cholesterol (mg/dL)	60.0 (48.0–72.0)	69	61.0 (53.0–71.0)	69	0.3036
LDL cholesterol (mg/dL)	109 ± 32	69	108 ± 28	69	0.8767
Non-HDL cholesterol (mg/dL)	129 ± 36	69	126 ± 29	69	0.5539
Homocysteine (µmol/L)	11.0 (9.0–13.0)	67	10.0 (9.0–13.0)	65	0.5781
Interleukin-6 (pg/mL)	1.55 (1.40–2.70)	66	1.50 (1.40–3.00)	65	0.9824
High-sensitivity CRP (mg/L)	1.17 (0.53–3.77)	68	0.84 (0.48–2.24)	66	0.0925
Ferritin (ng/mL)	48.5 (24.8–103)	68	58.0 (29.8–109)	68	0.3734
Serum folate (ng/mL)	6.4 (5.1–8.3)	68	7.9 (6.0–10.1)	69	0.0775
Alanine aminotransferase (U/L)	19.0 (14.0–25.0)	68	19.0 (14.0–26.0)	69	0.9416
Aspartate aminotransferase (U/L)	21.0 (19.0–25.0)	68	22.0 (18.0–25.0)	69	0.7841
Gamma-glutamyltransferase (U/L)	20.0 (14.8–28.0)	68	19.0 (14.0–26.0)	69	0.6791
Alkaline phosphatase (U/L)	67.5 (53.3–83.3)	68	68.0 (55.0–82.0)	69	0.9348
CKD-EPI eGFR < 90 mL/min/1.73 m^2^ (*n* (%))	14 (20.9%)	67	17 (25.0%)	68	0.5708
Hypertension history (*n* (%))	11 (15.9%)	69	8 (11.6%)	69	0.4586
Leukocytes (×10^3^/µL)	6.0 (5.4–7.4)	69	5.5 (4.7–6.5)	68	**0.0026**
Platelets (×10^3^/µL)	242 (209–276)	69	238 (206–282)	68	0.6778
Fibrinogen (mg/dL)	457 (382–512)	67	426 (379–507)	68	0.3908

Values are median (IQR), mean ± SD, or *n* (%), as indicated. Participants were classified according to the median legume intake of 18.558 g/day: low intake, <18.558 g/day; high intake, >18.558 g/day. No participant had an intake equal to the median. Statistically significant *p* values are shown in bold. CRP, C-reactive protein; eGFR, estimated glomerular filtration rate; HDL, high-density lipoprotein; HOMA-IR, homeostatic model assessment of insulin resistance; LDL, low-density lipoprotein.

**Table 3:** Cross-validated performance for estimating and discriminating the FFQ-derived legume-intake variable.

Model	RMSE, g/day	MAE, g/day	CV R^2^	Spearman ρ	Calibration Slope	AUC	Brier Score	Balanced Accuracy
Intercept-only benchmark	15.79 (15.72–15.93)	11.45 (11.38–11.57)	−0.015 (−0.033–−0.006)	—	—	0.500 (0.500–0.500)	0.254 (0.251–0.258)	0.500 (0.500–0.500)
Covariate model	15.67 (15.47–16.00)	11.11 (10.91–11.35)	0.001 (−0.042–0.026)	0.227 (0.174–0.276)	0.505 (0.250–0.655)	0.534 (0.497–0.563)	0.257 (0.251–0.264)	0.534 (0.478–0.569)
Biomarker model	15.19 (15.05–15.40)	10.81 (10.68–10.98)	0.061 (0.035–0.078)	0.305 (0.270–0.329)	0.797 (0.670–0.882)	0.653 (0.633–0.667)	0.236 (0.232–0.242)	0.619 (0.590–0.645)
Combined model	15.26 (15.06–15.58)	10.83 (10.65–11.13)	0.052 (0.012–0.077)	0.330 (0.290–0.363)	0.664 (0.538–0.748)	0.629 (0.605–0.653)	0.244 (0.238–0.252)	0.612 (0.580–0.638)

Values are means with empirical 2.5th–97.5th percentiles across 100 repetitions of 10-fold cross-validation. AUC refers to discrimination above versus at or below the sample median. RMSE, root-mean-square error; MAE, mean absolute error; CV R^2^, cross-validated coefficient of determination; AUC, area under the receiver-operating-characteristic curve. Rank correlation and calibration slope are not interpretable for the intercept-only benchmark because its predictions are effectively constant. For the intercept-only benchmark, AUC and balanced accuracy were set to 0.500 by definition because the model contains no discriminatory predictors. The percentile ranges describe variation across repeated resampling and should not be interpreted as conventional confidence intervals based on independent observations.

## Data Availability

The data presented in this study are available from the corresponding author upon reasonable request.

## Supplementary Materials

The following supporting information can be downloaded at: https://www.mdpi.com/article/10.3390/foods15183279/s1, Figure S1: Sensitivity analyses of the adjusted association between urinary hypaphorine and leukocyte count; Figure S2: Exploratory cross-sectional mediation model linking habitual legume intake, urinary hypaphorine, and leukocyte count; Table S1: Complete plant-based dietary correlation results for urinary hypaphorine, including Benjamini-Hochberg FDR correction across the seven dietary variables; Table S2: Multivariable models of urinary hypaphorine and leukocyte count; Table S3: Complete clinical and biochemical correlation results for urinary hypaphorine, including Benjamini-Hochberg FDR correction and the sensitivity analysis excluding >ULOQ observations; Table S4: Sensitivity analyses for the adjusted association between log10 urinary hypaphorine and leukocyte count; Table S5: Exploratory cross-sectional mediation estimates.
