# Supplementary material for: Urinary Hypaphorine as a Candidate Marker of FFQ-Derived Legume Intake Within the EAT-Lancet Framework in Free-Living Adults
Source: Foods. 2026 Sep 17;15(18):3279. doi: 10.3390/foods15183279 (PMC13606740; doi:10.3390/foods15183279)
Supplement: Supplementary file 1 [file foods-15-03279-s001.zip › foods-4497296-supplementary.pdf]

Supplementary Materials

Supplementary Figures

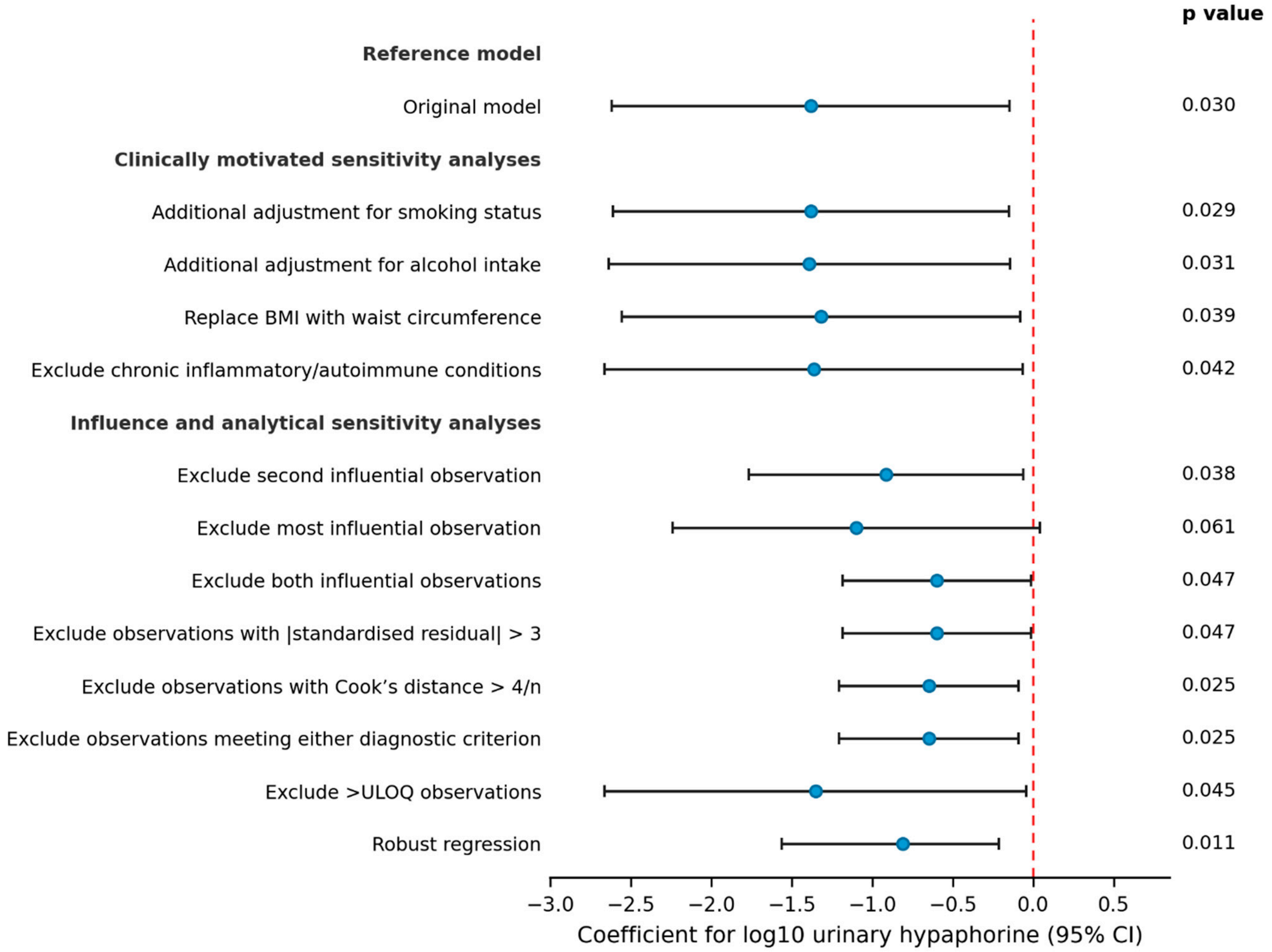

Figure S1. Sensitivity analyses of the adjusted association between urinary hypaphorine and leukocyte count. Points represent unstandardised coefficients for log10 urinary hypaphorine, and horizontal lines represent 95% confidence intervals. The figure includes the reference model, clinically motivated sensitivity analyses, and influence and analytical sensitivity analyses; full numerical results are provided in Table S4.

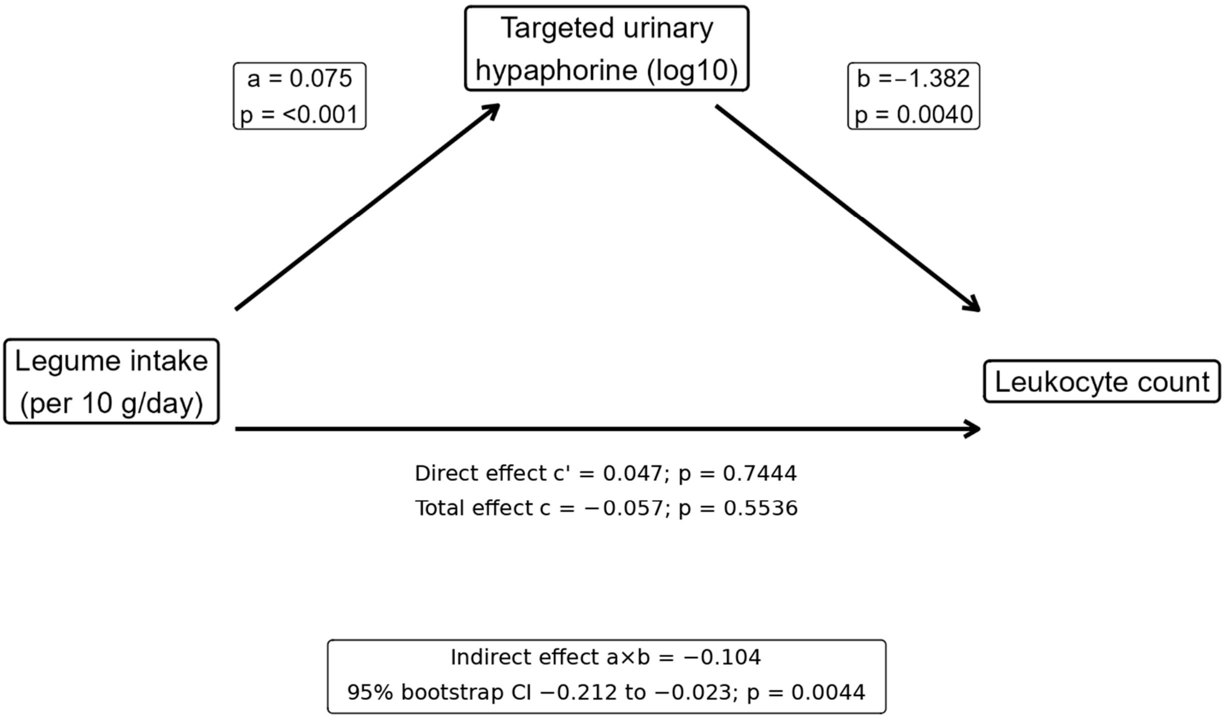

Figure S2. Exploratory cross-sectional mediation model linking habitual legume intake, urinary hypaphorine, and leukocyte count. Models were adjusted for age, sex, BMI, total energy intake, and the log10 urinary creatinine signal. The indirect effect was evaluated using 5000 nonparametric bootstrap resamples.

Supplementary Tables

Table S1. Complete plant-based dietary correlation results for urinary hypaphorine, including Benjamini-Hochberg FDR correction across the seven dietary variables.

Variable	n	Spearman rho	p value	BH-FDR q	n excluding >ULOQ	rho excluding >ULOQ	p excluding >ULOQ
Legume intake	138	0.240	0.0047	0.0326	134	0.219	0.011
Fruits	138	0.160	0.0616	0.1873	134	0.156	0.073
Whole grains	138	0.149	0.0803	0.1873	134	0.137	0.115
EAT-Lancet score	138	0.112	0.1912	0.3346	134	0.113	0.192
Combined plant-food intake	138	0.093	0.2783	0.3896	134	0.077	0.379
Nuts	138	0.080	0.3515	0.4101	134	0.065	0.457
Vegetables	138	−0.039	0.6528	0.6528	134	−0.038	0.661

Table S2. Multivariable models of urinary hypaphorine and leukocyte count. Coefficients are unstandardised, and confidence intervals use HC3 heteroscedasticity-consistent standard errors. In section B, the hypaphorine coefficient represents the difference in leukocyte count per tenfold higher urinary hypaphorine concentration.

**Outcome and term**	**Estimate**	**HC3 95% CI**	**p value**
A. Outcome: log10 urinary hypaphorine			
Intercept	−20.78352	−29.06468 to −12.50236	<0.001
Legume intake, g/day	0.00757	0.00304 to 0.01211	0.0014
Age, years	0.00173	−0.00493 to 0.00838	0.6121
Sex (female)	0.12351	−0.03989 to 0.28691	0.1409
BMI, kg/m²	−0.00413	−0.01667 to 0.00841	0.5197
Total energy intake, kcal/day	0.00011	0.00001 to 0.00022	0.0319
Log10 urinary creatinine signal	2.15862	1.27387 to 3.04337	<0.001
B. Outcome: leukocyte count (×10³/µL)			
Intercept	−31.33064	−67.05759 to 4.39630	0.0881
Log10 urinary hypaphorine	−1.38198	−2.61763 to −0.14633	0.0302
Legume intake, g/day	0.00468	−0.02164 to 0.03100	0.7280
Age, years	0.00778	−0.01784 to 0.03341	0.5527
Sex (female)	−0.22134	−1.00470 to 0.56202	0.5807
BMI, kg/m²	0.05272	0.00606 to 0.09938	0.0285
Total energy intake, kcal/day	−0.00003	−0.00050 to 0.00044	0.9036
Log10 urinary creatinine signal	3.83103	0.02785 to 7.63421	0.0505

Table S3. Complete clinical and biochemical correlation results for urinary hypaphorine, including Benjamini-Hochberg FDR correction and the sensitivity analysis excluding >ULOQ observations.

Variable	n	Spearman rho	Nominal p	BH-FDR q	n excluding >ULOQ	rho excluding >ULOQ	p excluding >ULOQ	q excluding >ULOQ
TG	137	−0.228	0.0074	0.0897	133	−0.244	0.0046	0.1105
WBC	137	−0.228	0.0075	0.0897	133	−0.208	0.0164	0.1964
α-Toc	120	−0.162	0.0773	0.6188	116	−0.158	0.0903	0.7222
GGT	137	−0.104	0.2281	0.8688	133	−0.081	0.3540	0.9403
ALT	137	−0.089	0.3001	0.8688	133	−0.056	0.5217	0.9403
TC	138	−0.069	0.4200	0.8688	134	−0.074	0.3980	0.9403
HOMA-IR	134	−0.063	0.4708	0.8688	130	−0.076	0.3886	0.9403
Ins	134	−0.060	0.4929	0.8688	130	−0.073	0.4100	0.9403
HDL-C	138	−0.058	0.4988	0.8688	134	−0.068	0.4330	0.9403
non-HDL-C	138	−0.056	0.5143	0.8688	134	−0.056	0.5208	0.9403
Hcy	132	−0.048	0.5879	0.8688	128	−0.050	0.5752	0.9403
Fib	135	−0.047	0.5904	0.8688	131	−0.019	0.8330	0.9504
25(OH)D	136	−0.046	0.5965	0.8688	132	−0.014	0.8712	0.9504
Plt	137	−0.031	0.7185	0.8688	133	−0.055	0.5300	0.9403
hs-CRP	134	−0.023	0.7887	0.8688	130	0.001	0.9865	0.9865
Ferritin	136	−0.023	0.7890	0.8688	132	−0.041	0.6375	0.9403
LDL-C	138	−0.018	0.8326	0.8688	134	−0.017	0.8423	0.9504
IL-6	131	0.012	0.8886	0.8886	127	0.039	0.6660	0.9403
Folate	137	0.021	0.8114	0.8688	133	0.031	0.7214	0.9504
HbA1c	136	0.027	0.7584	0.8688	132	0.038	0.6659	0.9403
Glucose	138	0.027	0.7528	0.8688	134	0.018	0.8378	0.9504
eGFR	135	0.034	0.6961	0.8688	131	0.005	0.9540	0.9865
AST	137	0.053	0.5353	0.8688	133	0.090	0.3051	0.9403
ALP	137	0.079	0.3564	0.8688	133	0.065	0.4589	0.9403

25(OH)D, 25-hydroxyvitamin D; ALP, alkaline phosphatase; ALT, alanine aminotransferase; AST, aspartate aminotransferase; eGFR, estimated glomerular filtration rate; Fib, fibrinogen; GGT, gamma-glutamyltransferase; HbA1c, glycated haemoglobin; Hcy, homocysteine; HDL-C, high-density lipoprotein cholesterol; HOMA-IR, homeostatic model assessment of insulin resistance; hs-CRP, high-sensitivity C-reactive protein; IL-6, interleukin-6; Ins, insulin; LDL-C, low-density lipoprotein cholesterol; non-HDL-C, non-high-density lipoprotein cholesterol; Plt, platelet count; TC, total cholesterol; TG, triglycerides; WBC, white blood cell count; α-Toc, α-tocopherol.

Table S4. Sensitivity analyses for the adjusted association between log10 urinary hypaphorine and leukocyte count. HC3 inference was used for ordinary least-squares models, whereas bootstrap inference was used for robust regression. Clinically motivated sensitivity analyses additionally adjusted for smoking status or alcohol intake, replaced BMI with waist circumference, or excluded participants with recorded chronic inflammatory or autoimmune conditions.

Analysis	n	Estimate	SE	95% CI lower	95% CI upper	p value
Original model	137	−1.3820	0.6304	−2.6176	−0.1463	0.0302
**Clinically motivated sensitivity analyses**						
Additional adjustment for smoking status	137	−1.3805	0.6272	−2.6098	−0.1512	0.0295
Additional adjustment for alcohol intake	137	−1.3905	0.6362	−2.6374	−0.1437	0.0306
Replace BMI with waist circumference	135	−1.3165	0.6315	−2.5541	−0.0788	0.0391
Exclude chronic inflammatory/autoimmune conditions	127	−1.3638	0.6618	−2.6610	−0.0667	0.0415
**Influence and analytical sensitivity analyses**						
Exclude second influential observation	136	−0.9130	0.4349	−1.7653	−0.0607	0.0377
Exclude most influential observation	136	−1.0984	0.5819	−2.2389	0.0420	0.0613
Exclude both influential observations	135	−0.5986	0.2990	−1.1848	−0.0125	0.0474
Exclude observations with |standardised residual| > 3	135	−0.5986	0.2990	−1.1848	−0.0125	0.0474
Exclude observations with Cook's distance > 4/n	127	−0.6487	0.2849	−1.2070	−0.0904	0.0246
Exclude observations meeting either diagnostic criterion	127	−0.6487	0.2849	−1.2070	−0.0904	0.0246
Exclude >ULOQ observations	133	−1.3518	0.6684	−2.6619	−0.0417	0.0453
Robust regression	137	−0.8096	0.3401	−1.5630	−0.2133	0.0110

Note: Smoking status was modelled as non-smoker, ex-smoker, or smoker. Alcohol intake was modelled continuously in g/day. Waist circumference replaced BMI in the central-adiposity sensitivity analysis. The inflammatory/autoimmune sensitivity excluded 10 participants with clearly identifiable chronic inflammatory or autoimmune conditions, including rheumatoid or inflammatory arthritis, multiple sclerosis, coeliac disease, autoimmune gastritis, eosinophilic oesophagitis, and sarcoidosis.

Table S5. Exploratory cross-sectional mediation estimates. Legume intake was scaled per 10 g/day. Confidence intervals and p values were obtained from 5000 nonparametric case-bootstrap resamples.

**Effect**	**n**	**Estimate**	**Bootstrap SE**	**Bootstrap CI lower**	**Bootstrap CI upper**	**Bootstrap p**
Path a: legume intake → log10 hypaphorine	137	0.0750	0.0222	0.0375	0.1259	<0.001
Path b: log10 hypaphorine → leukocytes	137	−1.3820	0.5977	−2.6316	−0.3137	0.0040
Indirect effect: a × b	137	−0.1036	0.0481	−0.2123	−0.0233	0.0044
Direct effect: c'	137	0.0468	0.1194	−0.2053	0.2670	0.7444
Total effect: c	137	−0.0568	0.1104	−0.2870	0.1408	0.5536
